# Development of a Fast and Robust UHPLC Method for Apixaban In-Process Control Analysis

**DOI:** 10.3390/molecules26123505

**Published:** 2021-06-08

**Authors:** Róbert Kormány, Norbert Rácz, Szabolcs Fekete, Krisztián Horváth

**Affiliations:** 1 Drug Substance Analytical Development Division, Egis Pharmaceuticals Plc., Keresztúri út 30-38, H-1106 Budapest, Hungary; kormany.robert@egis.hu (R.K.); rnorbi55@gmail.com (N.R.); 2 Waters Corporation, CMU-Rue Michel Servet 1, 1211 Geneva 4, Switzerland; Szabolcs_Fekete@waters.com; 3 Department of Analytical Chemistry, University of Pannonia, Egyetem Utca 10, H-8200 Veszprém, Hungary

**Keywords:** apixaban, design of experiments, liquid chromatography, method development, quality by design, robustness

## Abstract

In-process control (IPC) is an important task during chemical syntheses in pharmaceutical industry. Despite the fact that each chemical reaction is unique, the most common analytical technique used for IPC analysis is high performance liquid chromatography (HPLC). Today, the so-called “Quality by Design” (QbD) principle is often being applied rather than “Trial and Error” approach for HPLC method development. The QbD approach requires only for a very few experimental measurements to find the appropriate stationary phase and optimal chromatographic conditions such as the composition of mobile phase, gradient steepness or time ($t_{G}$), temperature (*T*), and mobile phase pH. In this study, the applicability of a multifactorial liquid chromatographic optimization software was studied in an extended knowledge space. Using state-of-the-art ultra-high performance liquid chromatography (UHPLC), the analysis time can significantly be shortened. By using UHPLC, it is possible to analyse the composition of the reaction mixture within few minutes. In this work, a mixture of route of synthesis of apixaban was analysed on short narrow bore column (50 × 2.1 mm, packed with sub-2 µm particles) resulting in short analysis time. The aim of the study was to cover a relatively narrow range of method parameters ($t_{G}$, *T*, pH) in order to find a robust working point (zone). The results of the virtual (modeled) robustness testing were systematically compared to experimental measurements and Design of Experiments (DoE) based predictions.

## 1. Introduction

The demands for enhancing the speed of method development are continuously growing nowadays. One needs to work out separation methods that are reliable and robust as well as time and solvent efficient. In order to meet all these necessary requirements, one could use Quality by Design (QbD) principles [1]. The conception of QbD is spreading not only through pharmaceutical production but also through the establishment of analytical methods [2].

In the synthesis of active pharmaceutical ingredients (API), analytical support is very important. The separation of API and its impurities is a necessary step in the control of pharmaceutical products. In many cases, the chemical structure of the impurities is quite different compared to the API. In most cases, reversed-phase liquid chromatography (RPLC) is the method of choice to analyze those API samples. In RPLC conditions, several parameters influence the quality of the separation. One of them is the stationary phase, which contributes to the separation by many different types of possible solute-phase interactions such as hydrophobic and electrostatic interactions as well as steric effects and H-bonding [3]. The other important parameters are the composition of the mobile phase (%B or time of gradient, $t_{G}$), pH, and temperature among others [4].

Intelligent systems (software) can assist to develop liquid chromatographic methods. One can obtain important information (such as solubility, the necessity of pH control, hydrophobicity, etc.) about the substance (e.g., p$K_{a}$ and log*P* values). There are several products on the market which are capable of predicting these data [5].

In this work, a new method has been developed for the mixture of synthesis route for apixaban using multifactorial optimization and modeling software of three measured method parameters, i.e., gradient time ($t_{G}$), temperature (T), pH, and 3 additional calculated parameters, i.e., the flow rate, %B_start_ and %B_end_, using UHPLC [6,7]. A robust and optimized analytical method is important also for in-process control (IPC). As far as we know, this is the first application of DryLab by an extended knowledge space for this purpose in the literature.

Apixaban is a compound being investigated as an anticoagulant which molecule is developed in a joint venture by Pfizer and Bristol-Myers Squibb and approved in the E.U. in 2011 for the prevention of venous thromboembolic events in adult patients, who have undergone elective hip or knee replacement [8]. One possible solution for the preparation of apixaban is described in the patent described by Egis Pharmaceuticals Plc [9].

## 2. Results

### 2.1. Sample

**Figure 1:**
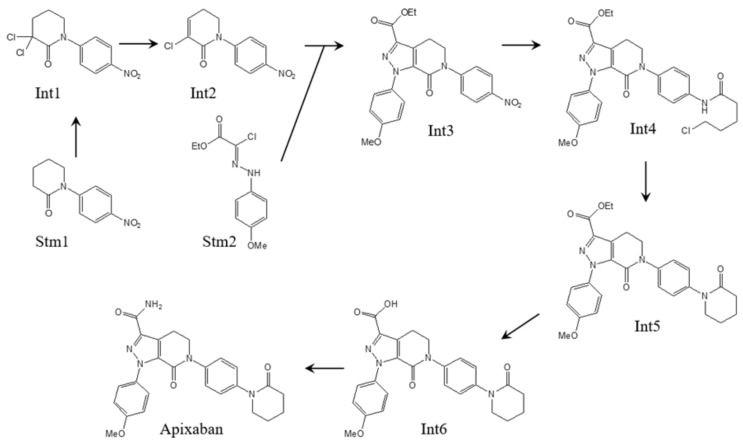
Flowchart of the apixaban synthesis.

Apixaban and its intermediates were used as test solutes, which were formed during the manufacturing process [9]. The flow chart of the synthesis is presented on Figure 1.

During the elaboration of API synthesis, not only the purity of the final product needs to be determined, but the reaction of the intermediates should also be monitored. At this stage of drug development, a fast analytical method is needed with which all intermediates can be analyzed.

For quick evaluation of the API quality, the peak area% is normally used. Since the specific UV absorption of the substances might be different, the correction (response) factors must be known for each compound at a given wavelength. To determine the correction factors, the intermediates were injected individually running a generic linear gradient method (10% ACN–80% ACN) and detected at 280 nm. The obtained correction factors are listed in Table 1.

### 2.2. Liquid Chromatographic Method Development

An extended experimental optimization strategy was used to develop the synthesis supporting the UHPLC method [2]. Drylab software was used to assist the method optimization procedure. The use of so-called 3D retention models (based on the simultaneous optimization of 3 method variables) is already well-established [6,7,10,11,12]. Assuming linear retention models for gradient steepness ($t_{G}$) and temperature (*T*), and non-linear model for mobile phase pH require a 12 experiment-based design.

The planned experimental space covered a temperature range of 60 ${}^{\circ }$C (20 ${}^{\circ }$C to 80 ${}^{\circ }$C) and 3.6 pH unit interval (2.8 to 6.4). To realize very fast method development, indeed steep gradients ($t_{G1}$ = 1.5 min, $t_{G2}$ = 4.5 min / 10%B→ 80%B), and high flow rate (0.8 mL/min) were set. Such flow rate can be applied at 20 ${}^{\circ }$C without reaching the upper limit of operating pressure (∼1000 bar). The concentration of the sample for each component was 10 µg/mL, the injection volume was 1 µL, and the detection was carried out at 280 nm.

10 mM citrate buffer was used as aqueous phase. Citric acid was chosen because it provides high enough buffer capacity between pH 2.8 and 6.4 (p$K_{1}$ = 3.1, p$K_{2}$ = 4.7 and p$K_{3}$ = 5.4). The appropriate pH was adjusted with additional sodium hydroxide solution.

At 80 ${}^{\circ }$C and pH = 6.4 Stm2 might be degraded, therefore, the planned experimental space could not be implemented. Two “partially extended” experimental spaces were examined. Parameters of the two experiments:

- $t_{G1}$ = 1.5 min; $t_{G2}$ = 4.5 min / $T_{1}$ = 20 ${}^{\circ }$C; $T_{2}$ = 80 ${}^{\circ }$C / pH${}_{1}$ = 2.8; pH${}_{2}$ = 4.0; pH${}_{3}$ = 5.2
- $t_{G1}$ = 1.5 min; $t_{G2}$ = 4.5 min / $T_{1}$ = 20 ${}^{\circ }$C; $T_{2}$ = 50 ${}^{\circ }$C / pH${}_{1}$ = 2.8; pH${}_{2}$ = 4.6; pH${}_{3}$ = 6.4

**Figure 2:**
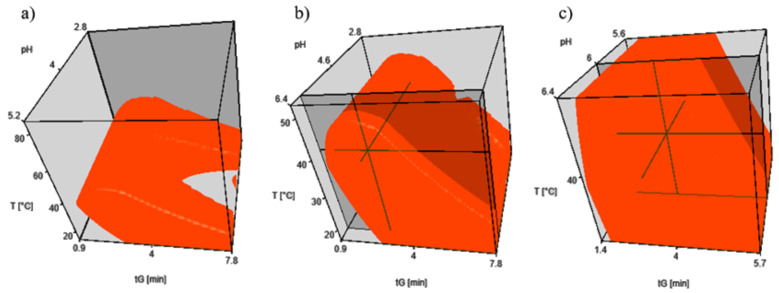
Design Spaces in the 3D models. The irregular red zones indicate the Design Spaces of the UHPLC-method, where the critical resolution is higher than 2.0. (**a**) $t_{G1}$ = 1.5 min; $t_{G2}$ = 4.5 min/$T_{1}$ = 20 ${}^{\circ }$C; $T_{2}$ = 80 ${}^{\circ }$C/pH${}_{1}$ = 2.8; pH${}_{2}$ = 4.0; pH${}_{3}$ = 5.2; (**b**) $t_{G1}$ = 1.5 min; $t_{G2}$ = 4.5 min/$T_{1}$ = 20 ${}^{\circ }$C; $T_{2}$ = 50 ${}^{\circ }$C/pH${}_{1}$ = 2.8; pH${}_{2}$ = 4.6; pH${}_{3}$ = 6.4; (**c**) $t_{G1}$ = 2.0 min; $t_{G2}$ = 4.0 min/$T_{1}$ = 35 ${}^{\circ }$C; $T_{2}$ = 45 ${}^{\circ }$C/pH${}_{1}$ = 5.8; pH${}_{2}$ = 6.0; pH${}_{3}$ = 6.4.

After measuring the experimental points, retention models were created (Figure 2a,b). Please note, that in the design spaces only the ranges are indicated where the critical resolution met the $R_{S,\mathrm{crit}}>$ 2.0 criterion. A higher resolution criterion was chosen than is required for baseline separation ($R_{S}$ = 1.5) because in practice there may be a difference of 2–3 orders of magnitude in the sample concentration of the substances to be tested. The lower resolution of the critical peak pair may make it difficult to quantify the closely eluting compounds.

The result of the first modeling is illustrated in Figure 2a. The figure shows that high temperature (>50 ${}^{\circ }$C) - in this case - does not favor the separation, the minimum resolution criterion is not met, therefore it is justified to perform the second model experiment, the results of which are illustrated in Figure 2b. In the pH range of 2.8 to 4.0, apixaban and Int6 are difficult to separate, and in the range of 2.8 to 5.2, the retention of the acidic Int6 is strongly pH-dependent, i.e. no robust method can be developed. When selecting the working point, it is advisable to look for parameters that are far from the applicability limit (edge of the red range), and at the same time do not result in lengthy analysis. The parameters $t_{G}$ = 3.0 min (10%B → 80%B), T = 40 ${}^{\circ }$C, pH = 6.0 meet these criteria. A small part of the space around the working point in Figure 2b was cut out, on which the robustness test was performed (Figure 2c). The chromatograms required to construct the model in Figure 2c were exported from the model in Figure 2b.

**Figure 3:**
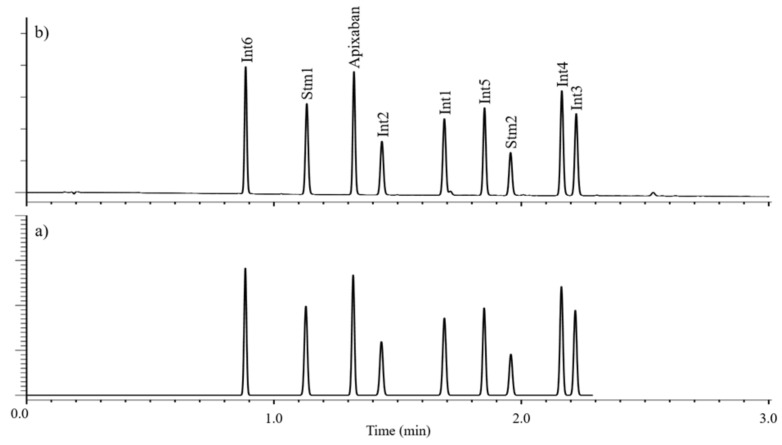
Simulated (**a**) and measured (**b**) chromatograms.

**Figure 4:**
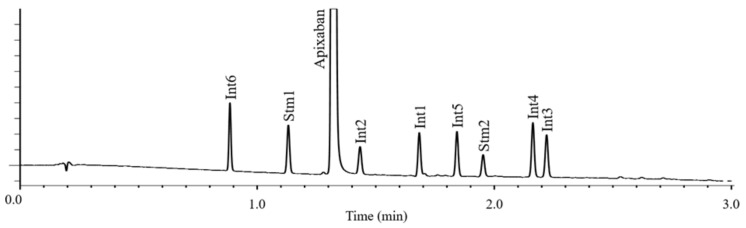
Chromatogram of apixaban solution spiked at 0.1% level.

The required time for method development was 2 workdays (16 h), and only 400 mL eluent was used. The analysis time is 3 min, so in addition to the final qualification, the test method can be used for both preparative research and in-process control (IPC). Calculated and experimentally measured chromatograms are shown in Figure 3 while Figure 4 was obtained by injecting a 1 mg/mL spiked solution (at 0.1% level).

### 2.3. Robustness Testing

A virtual robustness testing was performed using the robustness module of DryLab. In addition to the three measured model parameters ($t_{G}$, *T*, pH), the flow rate, the initial %B composition, and final %B compositions of the mobile phase gradient were introduced as calculated parameters and included in the model (6 parameters in total). The effects of these 6 parameters are calculated, optionally at 2 or 3 levels. In our case, we studied them at 3 levels.

Software-based robustness calculation has the advantage that not only all solvent and instrument effects but also all conceivable combinations (parameter interactions) of them can be calculated in a model-mediated way [11]. The modeled deviations (−1, 0 and +1 factor levels) from the nominal values were set as: gradient time 2.7, 3.0 and 3.3 min, temperature to 38, 40 and 42 ${}^{\circ }$C, pH to 5.8, 6.0 and 6.2, flow rate to 0.72, 0.80 and 0.88 mL/min, initial mobile phase composition to 9, 10 and 11%B and its final composition to 79, 80 and 81%B. Then, the 729 experiments (36) were performed in silico.

**Figure 5:**
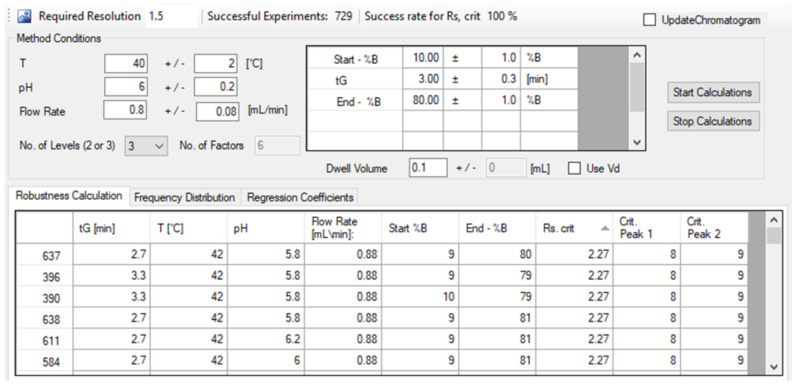
Set deviations (levels) of method parameters considered for the virtual robustness study and the calculated results ($R_{S,\mathrm{crit}}$ and critical peak pairs) for the 6 worst separations among the 729 virtual experiments.

A criterion of $R_{S,\mathrm{crit}}>2.0$ was considered. As shown is Figure 5, the lowest predicted resolution was $R_{S}$ = 2.27 between peak 8 and 9 (Int4 and Int3) which is still acceptable. Therefore, the method can be considered as robust, since the success rate to perform $R_{S,\mathrm{crit}}>2.0$ separation was 100% in the studied range of method parameters.

Very good agreement was observed between the predicted and measured retention times and resolutions (see Table 2).

## 3. Materials and Methods

The mobile phase was a mixture of acetonitrile and 5mM citrate buffer. Acetonitrile (ACN, gradient grade), citric acid, sodium hydroxide, standard reference buffers (pH 2.00, 4.01, and 7.00) were purchased from Merck (Darmstadt, Germany). For the measurements, water was prepared freshly using ELGA Purelab UHQ water (ELGA, Lane End, UK). The buffer was filtered before use on regenerated cellulose filter membrane, 0.2 µm pore size (Sartorius, Göttingen, Germany). Starting materials (Stm), intermediates (Int), and apixaban were purchased from Egis Pharmaceuticals Plc. chemical standard store (Budapest, Hungary). The concentrations of the samples were in the range of 0.01 to 0.5 mg/mL. The detector response was linear in this range. A mixture of water and ACN (50:50%) was used as a dissolution solvent.

UHPLC measurements were performed using a Waters Acquity UPLC system (Milford, CT, USA) equipped with a binary solvent delivery pump, an autosampler, a photodiode array detector, 5 µL injection loop, 500 nL flow cell and Empower software (version 3). The column was an Acquity BEH C18 50 × 2.1 mm, 1.7 µm. The dwell volume of the system was 0.12 mL.

The MP 225 pH-meter was purchased from Mettler-Toledo (Mettler-Toledo, Greifensee, Switzerland). Retention modeling was carried out using DryLab v.4.3.1 and the quantitative robustness evaluation of generated models was performed with the latest DryLab Robustness Module (Molnár-Institute, Berlin, Germany).

## 4. Conclusions

In this work, a new UHPLC method has been developed for the in-process control analysis of apixaban. Method development was supported by state-of-the-art chromatographic modeling software. The quality of the separation (resolution map) was studied in an extended knowledge space by combining three complementary design spaces. This approach enabled to model and study retention and peak resolution in a broad pH range (between pH 2.8 and 6.4). Besides pH, the impact of gradient steepness and temperature was studied as well. A very fast—3 min—linear gradient method was found to perform suitable separation for all compounds. Then method robustness was studied by performing a virtual robustness test (employing 729 virtual experiments). In the end, the method was experimentally verified and excellent agreement was found between calculated and measured chromatograms. 

## Figures and Tables

**Table 1:** Correction factors of apixaban and its intermediers, measured at 280 nm.

	Stm1	Int1	Int2	Stm2	Int3	Int4	Int5	Int6	Apixaban
Stm1	1.00	0.87	0.64	—	0.92	1.13	0.90	0.98	1.16
Int1	—	1.00	0.73	—	1.06	1.30	1.03	1.13	1.34
Int2	—	—	1.00	—	1.44	1.77	1.41	1.54	1.82
Stm2	—	—	—	1.00	1.88	2.32	1.84	2.01	2.38
Int3	—	—	—	—	1.00	1.23	0.98	1.07	1.27
Int4	—	—	—	—	—	1.00	0.79	0.87	1.03
Int5	—	—	—	—	—	—	1.00	1.09	1.29
Int6	—	—	—	—	—	—	—	1.00	1.19
Apixaban	—	—	—	—	—	—	—	—	1.00

**Table 2:** Differences between simulated and measured retention times (min) and resolutions when examined for parameters belonging to the operating point (OP) and the six worst resolutions (for parameters of the six scenarios, see Figure 5).

	OP		637		396		390		638		611		584	
	Pred.	Exp.	Pred.	Exp.	Pred.	Exp.	Pred.	Exp.	Pred.	Exp.	Pred.	Exp.	Pred.	Exp.
Int6	0.88	0.89	0.83	0.83	0.93	0.94	0.89	0.89	0.82	0.83	0.82	0.83	0.82	0.82
Stm1	1.13	1.13	1.05	1.04	1.16	1.16	1.12	1.12	1.04	1.03	1.04	1.04	1.04	1.03
Apixaban	1.32	1.32	1.22	1.21	1.40	1.40	1.36	1.36	1.21	1.22	1.21	1.22	1.21	1.21
Int2	1.43	1.44	1.31	1.31	1.48	1.46	1.44	1.42	1.30	1.30	1.30	1.32	1.30	1.31
Int1	1.69	1.69	1.54	1.55	1,76	1.75	1.72	1.72	1.53	1.53	1.53	1.53	1.53	1.53
Int5	1.85	1.85	1.69	1.67	1.96	1.98	1.93	1.94	1.67	1.68	1.67	1.68	1.67	1.67
Stm2	1.96	1.96	1.78	1.77	2.06	2.05	2.03	2.05	1.77	1.79	1.77	1.78	1.77	1.78
Int4	2.16	2.16	1.96	1.95	2.30	2.30	2.28	2.29	1.94	1.95	1.94	1.94	1.94	1.95
Int3	2.22	2.22	2.01	1.98	2.35	2.37	2.33	2.34	1.99	2.00	1.99	1.99	1.99	1.99
$R_{S,\mathrm{crit}}$ **Int4-Int3**	**2.40**	**2.37**	**2.27**	**2.24**	**2.27**	**2.25**	**2.27**	**2.24**	**2.27**	**2.23**	**2.27**	**2.23**	**2.27**	**2.23**

## Data Availability

The data presented in this study are available on request from the corresponding author.

## Sample Availability

Samples of the compounds are not available from the authors.

## Abbreviations

The following abbreviations are used in this manuscript:

DOAJ	Directory of open access journals
ACN	Acetonitrile
API	Active Pharmaceutical Ingredient
DoE	Design of Experiment
HPLC	High-Performance Liquid Chromatography
IPC	In-Process Control
QbD	Quality by Design
RPLC	Reversed Phase Liquid Chromatography
UHPLC	Ultra-High-Performance Liquid Chromatography
